# Prognostic, diagnostic and clinicopathological roles of tsRNAs: a meta-analysis in breast cancer

**DOI:** 10.1186/s40001-023-01617-2

**Published:** 2024-01-08

**Authors:** Lu-Jue Gao, Si-Xun Zhu, Ying-Yi Wei, Hua-Wei Meng, Jing Gu, Hao Zhang, Li-Juan Dai

**Affiliations:** 1 Taicang Hospital of Traditional Chinese Medicine, Suzhou, China; 2 Jiangyin Traditional Chinese Medicine Hospital, Jiangyin, China; 3 Nanjing University of Chinese Medicine, Nanjing, China

**Keywords:** Prognostic, Diagnostic, Clinicopathological, tsRNAs, Breast cancer, Meta

## Abstract

Breast cancer (BC) is one of the most common malignancies in women and the leading cause of cancer-related death in women. The newly emerged non-coding RNAs tsRNAs (tRNA-derived small RNAs) play an important role in the occurrence and development of BC. The purpose of this study was to comprehensively evaluate the prognostic, diagnostic and clinicopathological roles of tsRNAs in BC. Through literature screening, a total of 13 BC-related tsRNA studies were included in this meta-analysis, all of which passed quality assessment. Prognostic studies showed upregulated tsRNAs to be associated with poor survival outcomes (HR = 1.64, 95%CI 1.51–1.77) and downregulated tsRNAs to be associated with better outcomes (HR = 0.58, 95%CI 0.50–0.68). Results of diagnostic studies showed a combined sensitivity of 72% (95%CI 68–76%) and combined specificity of 64% (95%CI 61–67%); the AUC was 0.72 (95%CI 0.68–0.75) and the DOR 4.62 (95%CI 3.76–5.68). Finally, correlation analysis of clinicopathological features showed that downregulation of tsRNAs correlated significantly with age, TNM stage and lymphatic metastasis. Sensitivity analysis and publication bias showed no significant difference. In conclusion, BC-associated tsRNAs are closely related to the prognosis and clinicopathological features of patients with this disease and can be used to assist in early diagnosis of BC. Therefore, tsRNAs are potential targets for the diagnosis and treatment of BC.

**Supplementary Information:**

The online version contains supplementary material available at 10.1186/s40001-023-01617-2.

## Introduction

According to the latest cancer statistics data, breast cancer (BC) is the most common malignant tumor in women, accounting for 31% of the total number of female cancers, and the incidence has gradually increased in recent years [1, 2]. With continuous improvement in diagnosis and treatment of BC, early- stage patients have good prognosis, with an overall cure rate of 90% [3]. However, the 5-year survival rate is significantly reduced for patients with advanced stage, poor tissue typing or resistance to combined therapy [4]. Therefore, clarifying the specific molecular mechanism of the occurrence and development of BC to assist in early diagnosis, as well as to find new and more accurate targeted molecules, is crucial for improving the overall survival rate of BC patients.

In recent years, positive roles of a variety of non-coding RNAs (ncRNAs), including micro RNAs (miRNAs), long non-coding RNAs (lncRNAs), circular RNAs (circRNAs) and tRNA-derived small RNAs (tsRNAs), in cancer have received much attention and been widely reported; some have been included in clinical application, fully demonstrating the huge potential of ncRNAs in tumor diagnosis and treatment [5–7]. TsRNAs are the products of tRNA or pre-tRNA cleavage during maturation. TsRNAs can be divided according to different fracture sites into tRNA-related fragments (tRFs) and tRNA halves (tiRNAs), which complement each other in terms of formation pathway, cell localization and function [8, 9]. Although tsRNAs were initially thought to be produced by random degradation and have no special function, an increasing number of studies have shown that tsRNAs are not only widely expressed in tumors but also play important roles in the occurrence and development of tumors [10]. In addition, tsRNAs are present in a large number of human body fluids, such as saliva, urine, and semen, providing a new target for early tumor diagnosis and precise treatment [11].

Studies have shown that tsRNAs are expressed specifically in BC and widely exist in the serum of BC patients. TsRNAs differentially expressed in different subtypes of BC, including triple-negative breast cancer, have been mined through RNA sequencing and verified with clinical samples, suggesting the significance of tsRNAs in identifying tumor properties and tumor tissue types [12–14]. In terms of mechanism research, tsRNAs are significantly enriched in hormone-dependent BC and are not only directly involved in regulating the occurrence and development of BC but also closely related to drug resistance and tumor recurrence in BC [15–17]. Overall, tsRNAs are good detection markers and potential therapeutic targets for BC patients. Therefore, we summarized existing studies on BC and tsRNAs and used meta-analysis to explore the roles and values of tsRNAs in the prognosis, diagnosis and clinicopathological features of BC patients to provide evidence for future applications of tsRNAs.

## Methods

### Publication search

To collect relevant research literature for meta-analysis, the Boolean logic method was used to search PubMed, Web of Science, Cochrane Library, Embase and other databases as of March 1, 2023. Specific search terms included “breast cancer”, “breast carcinoma”, “tsRNA”, “tRNA-derived small RNA”, “tRF”, “tRNA-derived fragment”, “tiRNA” and “tRNA-derived stress-induced RNA”.

### Inclusion and exclusion criteria

Inclusion criteria: (1) the content included the relationship between tsRNAs and BC; (2) all cases passed the gold standard, that is, the histopathological diagnosis was clear; and (3) data related to prognosis, diagnosis or clinicopathological features could be extracted directly or indirectly. Exclusion criteria: (1) the content was not related to tsRNAs or BC; (2) reviews, meta-analyses, letters, case reports, conference abstracts; (3) non-English and nonhuman studies; and (4) failure to report or extract important indicators and data. Two researchers independently read the title and abstract of the retrieved literature for preliminary screening; in cases in which the abstract results were not clear, the full text was read to determine inclusion eligibility. If the screening results were inconsistent, the third researcher decided.

### Data extract

The general data extracted from each article included the following: title, first author, tsRNAs, year, country, expression, cut-off, sample size, data source, detected sample, among others. Data related to prognosis included follow-up time, survival outcome (progression-free survival (PFS), recurrence-free survival (RFS), disease-free survival (DFS), overall survival (OS)), survival analysis, hazard ratio (HR), and 95% confidence interval (95%CI), among others. Data related to diagnosis included case, control, area under the receiver operating characteristic curve (AUC), sensitivity (SEN), specificity (SPE), true positive (TP), false positive (FP), true negative (TN), false negative (FN), positive likelihood ratio (PLR), negative likelihood ratio (NLR), diagnostic odds ratio (DOR), and cancer type, among others. Data related to clinicopathologic features included age, TNM stage, and lymphatic metastasis, among others. All records were approved by two independent researchers.

### Quality assessment

As the quality of studies may influence the results of meta-analysis, each included study was evaluated using the Newcastle‒Ottawa score (NOS) tables in the Cochrane manual and Quality Assessment for Studies of Diagnostic Accuracy 2 (QUADAS 2). After all evaluations, RevMan 5.3 software was used to output the evaluation results. NOS scores ≥ 7 or QUADAS 2 scores ≥ 4 are generally considered to be high quality [18, 19].

### Data synthesis and analysis

Statistical analysis of the data was performed with Stata software (version 15.1). HRs, odds ratios (ORs), and 95%CIs were used to evaluate the effect of tsRNAs on prognosis and clinicopathologic features in BC patients. To evaluate the diagnostic value of tsRNAs, summary receiver operating characteristic (SROC) curves were drawn, and AUCs were calculated, as were Fagan nomograms and scatter plots. For studies in which Kaplan–Meier (KM) and ROC curve data could not be extracted directly, we used Engage Digitizer and GetData Graph Digitizer and calculated HR and the corresponding 95%CI based on the method of Tierney et al. [20–22]. The Cochran-Q test and *I*^2^ statistics were used to assess heterogeneity. When the heterogeneity was small (*P* > 0.10 or *I*^2^ < 50%), a meta-analysis was conducted using the fixed effects model. Otherwise, random-effects models were employed to combine effect sizes, and further subgroup analysis and meta-regression (MetaDiSc software) were applied to explore possible sources of interstudy heterogeneity. Sensitivity analysis was carried out by excluding the included studies one by one. Finally, Begg’s funnel plot, Deeks’ funnel plot and Egger’s test were used to evaluate publication bias, and *P* > 0.05 was considered to indicate no publication bias. All statistical tests were bilateral, and P < 0.05 was considered statistically significant.

## Results

### Literature information and study characteristics

**Fig. 1:**
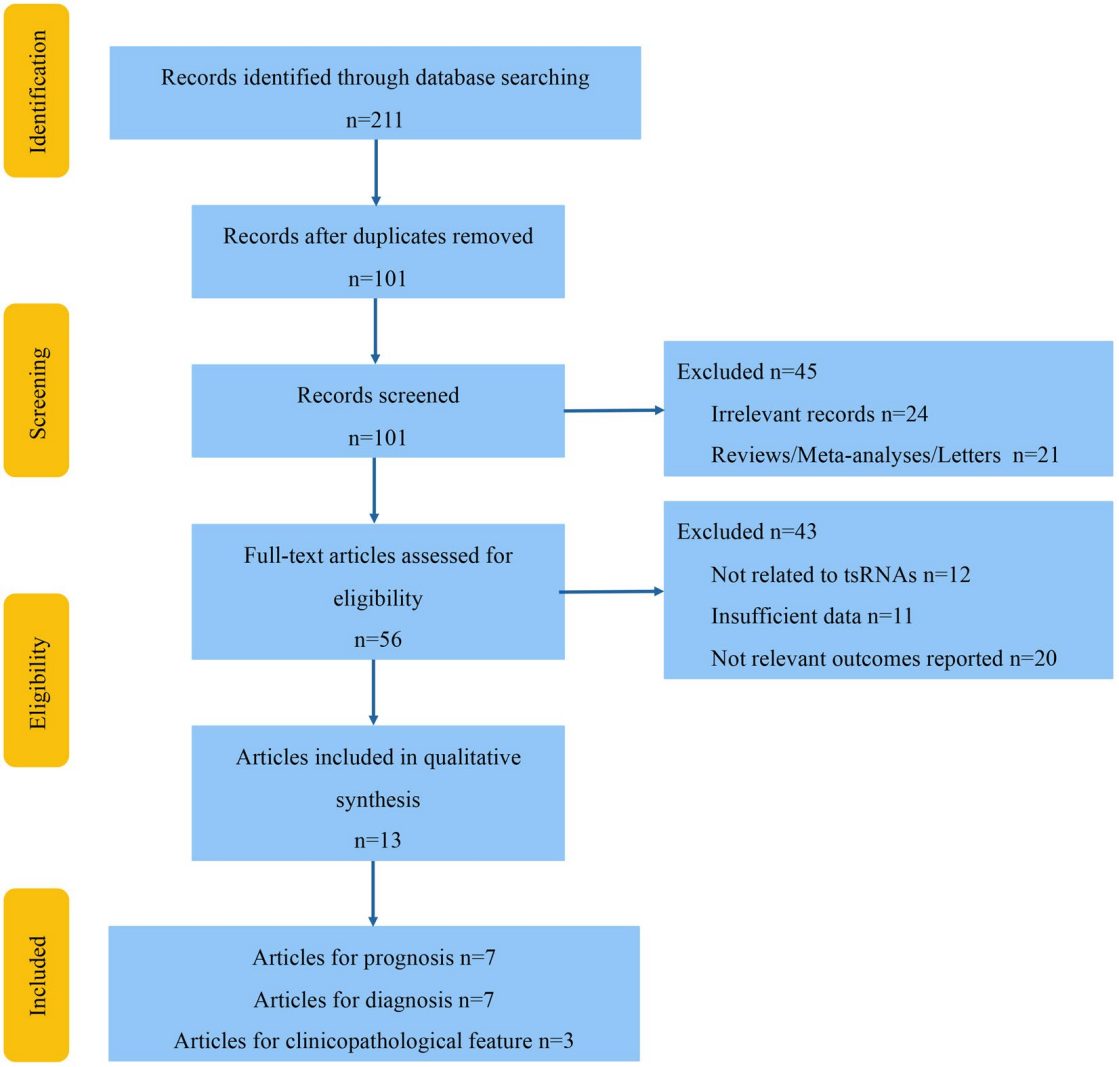
Workflow of the study and result of document screening

Through systematic analysis using our search terms, a total of 211 studies were retrieved; 101 remained after removing duplicate studies. Then, 45 studies that did not meet the inclusion criteria were excluded, and the full texts of the remaining 56 studies were reviewed in detail. Finally, 13 studies (involving 7 prognosis, 7 diagnosis, and 3 clinicopathological features) were included in the meta-analysis [13, 23–34]. The detailed literature selection process is illustrated in Fig. 1.

NOS and QUADAS 2 scoring methods were used to systematically assess the quality of all included prognostic and diagnostic studies. The results showed that the quality of all the included studies met the standards. Risk of bias graphs and summaries are shown in Additional file 1: Figures S1 and Additional file 2: Figure S2.

### Prognostic roles of tsRNAs in BC

There were seven studies, all of which were conducted in China except for one in the USA and published between 2018 and 2022. The sample type used for detection was mainly tissue, with a total of 5257 patients. Clinical outcome indicators included OS, PFS, RFS and DFS. Finally, 10 high expression tsRNAs and 6 low expression tsRNAs related to BC were identified (Table 1).

**Table 1:** Characteristics of all prognostic studies included in the meta-analysis

No	Study/TsRNA	Year	Country	Expression	Cut-off	Sample size	Data source	Detected sample	Follow-up time	Survival outcome	Survival analysis	Variables				Ref^c^
												HR^a^	95% CI^b^	*p*-value	Obtained	
1	Sun, tRF-30-JZOYJE22RR33	2018	China	Up	ΔCT	52	Clinical	Tissue	30	PFS^d^	Multe^i^	2.754	1.038–5.219	0.04	Direct	30153663
2	Sun, tRF-27-ZDXPHO53KSN	2018	China	Up	ΔCT	52	Clinical	Tissue	30	PFS	Multi	2.265	1.187–6.756	0.019	Direct	30153663
3	Feng, tDR-000620	2018	China	Down	Med^f^	44	Clinical	Plasma	40	RFS^g^	Multi	0.265	0.073–0.959	0.043	Direct	30239174
4	Shan, tRFdb-5024a	2020	China	Down	NA	1081	Database	Tissue	250	OS^h^	Uni^i^	0.52	0.37–0.74	< 0.001	Direct	32785169
5	Shan, 5P_tRNA-Leu-CAA-4–1	2020	China	Down	NA	1081	Database	Tissue	250	OS	Uni	0.55	0.35–0.87	0.011	Direct	32785169
6	Shan, ts-49	2020	China	Down	NA	1081	Database	Tissue	250	OS	Uni	0.4	0.17–0.93	0.032	Direct	32785169
7	Shan, ts-34	2020	China	Up	NA	1081	Database	Tissue	250	OS	Uni	1.62	1.08–2.44	0.019	Direct	32785169
8	Shan, ts-58	2020	China	Up	NA	1081	Database	Tissue	250	OS	Uni	1.56	1.1–2.2	0.013	Direct	32785169
9	Wang, tRF-Glu-CTC-003	2020	China	Down	NA	144	Clinical	Plasma	60	DFS^j^	Uni	0.37	0.04–3.57	0.144	KM^k^	32,814,252
10	Wang, tRF-Glu-CTC-003	2020	China	Down	NA	144	Clinical	Plasma	60	OS	Uni	0.9	0.1–8.29	0.1714	KM	32814252
11	Wang, tRF-Arg-CCT-017	2021	China	Up	Med	120	Clinical	Plasma	60	DFS	Uni	2.23	0.03–193.98	0.0123	KM	33402674
12	Wang, tRF-Arg-CCT-017	2021	China	Up	Med	120	Clinical	Plasma	60	OS	Uni	1.71	0.05–64.25	0.0428	KM	33402674
13	Zhang, tRNA-Lys-TTT-3–1	2022	China	Up	Med	1101	Database	Tissue	260	OS	Uni	1.48	1.24–1.76	0.028	KM	35030975
14	Zhang, tRNA-Gly-GCC-1–4	2022	China	Up	Med	1101	Database	Tissue	260	OS	Uni	1.64	1.37–1.95	0.043	KM	35030975
15	Zhang, tRNA- Ser-AGA-2–5	2022	China	Up	Med	1101	Database	Tissue	260	OS	Uni	2.24	1.83–2.74	0.0041	KM	35030975
16	Zhang, tRNA-Ser-AGA-3–1	2022	China	Up	Med	1101	Database	Tissue	260	OS	Uni	1.39	1.15–1.68	0.021	KM	35030975
17	Zhang, tRNA-His-GTG-1–1	2022	China	Down	Med	1101	Database	Tissue	260	OS	Uni	0.63	0.52–0.76	0.041	KM	35030975
18	Liu, 5'-tRF-Cys	2022	USA	Up	NA	978	Database	Tissue	120	OS	Multi	1.6	1.02–2.52	0.041	Direct	35654044
19	Liu, 5'-tRF-Cys	2022	USA	Up	NA	671	Database	Tissue	120	OS	Multi	1.76	1.02–3.04	0.04	Direct	35654044
20	Liu, 5'-tRF-Cys	2022	USA	Up	NA	1066	Database	Tissue	120	OS	Uni	1.57	1.23–2.02	0.011	KM	35654044

a HR: hazard ratio; b 95% CI 95% confidence interval; c Ref: reference; d PFS: progression-free survival; e Multi: multivariate; f Med: median; g RFS: recurrence-free survival; h OS: overall survival; i Uni: univariate; j DFS: disease-free survival; k KM: KM curve

**Fig. 2:**
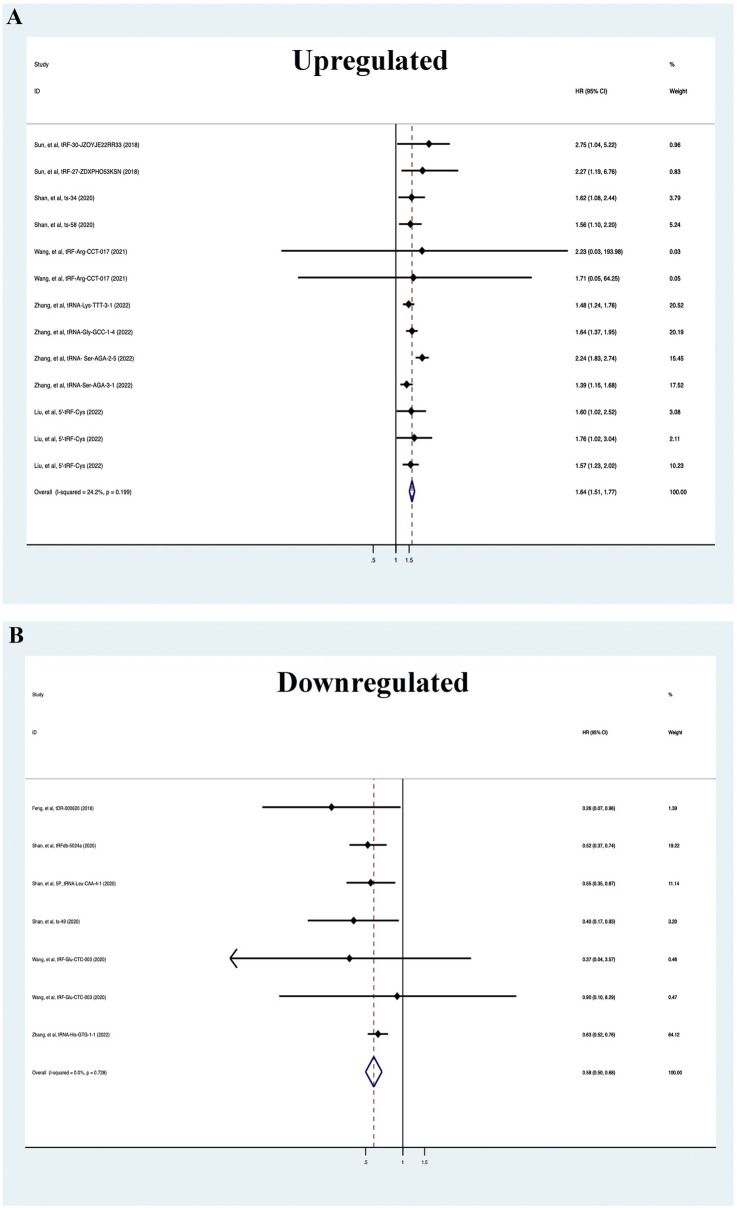
Forest plots of upregulated (**A**) and downregulated (**B**) tsRNAs for survival outcome in breast cancer

The fixed effect model was used to evaluate the correlation between tsRNAs and prognosis of BC patients according to the level of heterogeneity. According to forest map results, high expression of tsRNAs was associated with poor clinical outcomes (HR = 1.64, 95%CI 1.51–1.77). Conversely, low expression of tsRNAs was associated with better clinical outcomes (HR = 0.58, 95%CI 0.50–0.68). These results indicate that tsRNAs may be important prognostic factors for BC (Fig. 2). In addition, subgroup analysis of differentially expressed tsRNAs was performed, and the results showed that parameters such as country, cut-off, sample size, data source, detected sample, follow-up time, survival outcome, survival analysis, and HR obtained were associated with BC prognosis (Additional file 3: Figure S3 and Additional file 4: Figure S4).

### Diagnostic roles of tsRNAs in BC

**Fig. 3:**
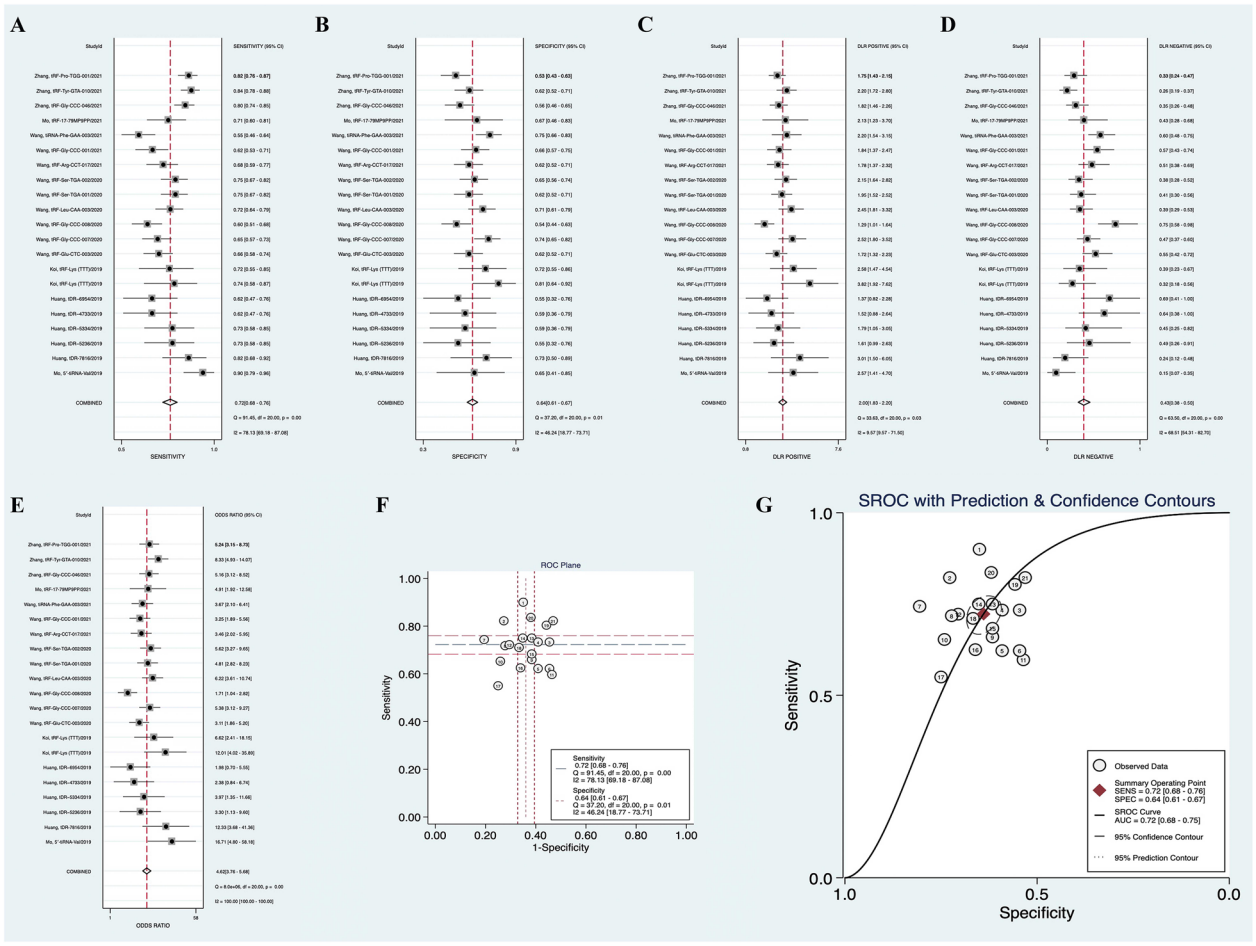
Forest plots of combined sensitivity (**A**), specificity (**B**), PLR (**C**), NLR (**D**), DOR (**E**), ROC plane (**F**), and SROC curve (**G**) of tsRNAs for breast cancer

**Fig. 4:**
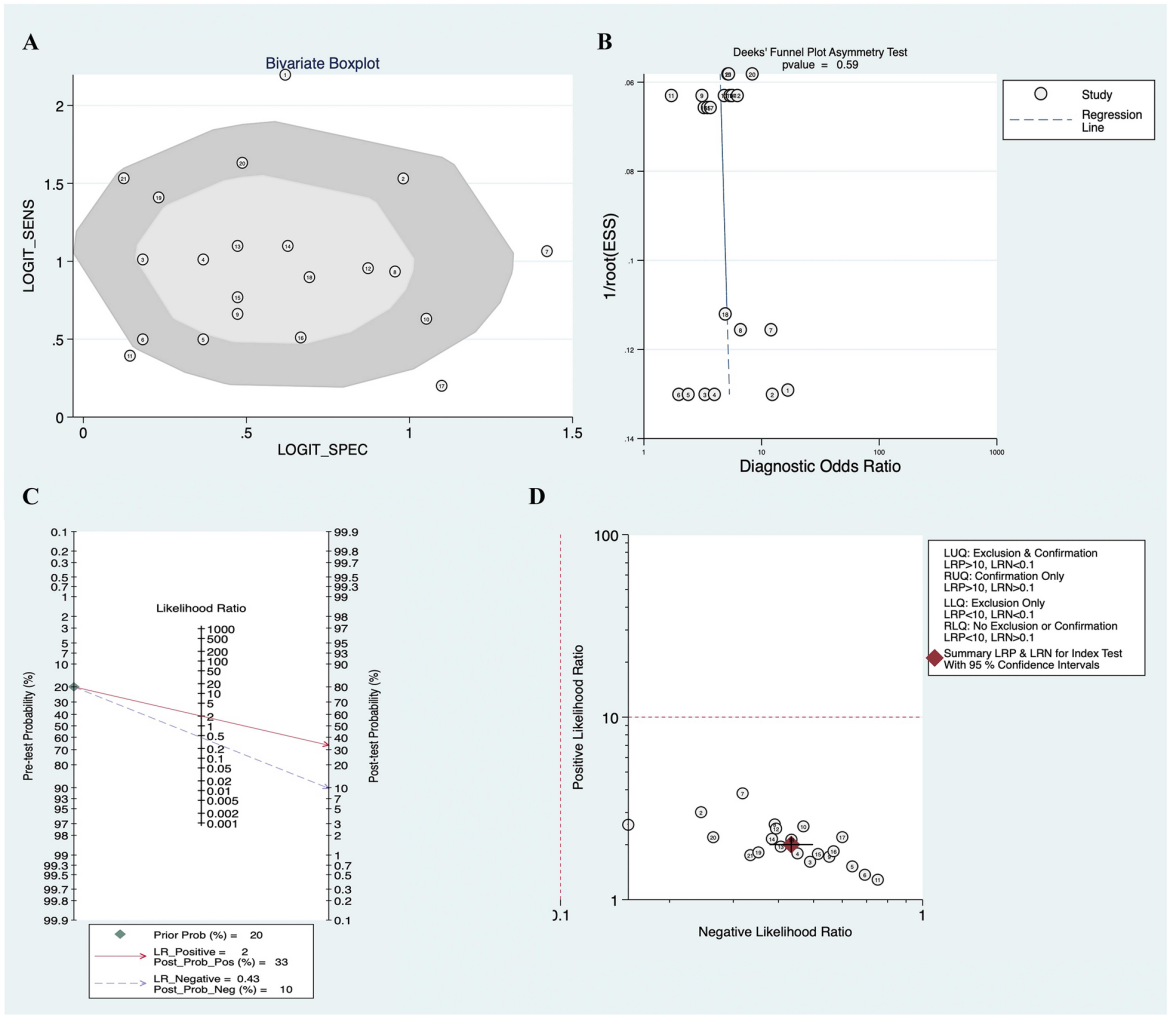
Bivariate boxplot (**A**), Deeks’ funnel plot (**B**), scatter plot of PLR and NLR (**C**), and Fagan's nomogram (**D**) of tsRNAs for breast cancer

In Table 2, the SEN, SEP, LPR, NPR and DOR of BC diagnosis are summarized, and the results are shown in the form of a forest map. The combined SEN (Fig. 3A) was 72% (95%CI 68–76%), and the combined SEP (Fig. 3B) was 64% (95%CI 61–67%). The combined PLR (Fig. 3C) was 2.00 (95%CI 1.83–2.20) and the combined NLR (Fig. 3D) 0.43 (95%CI 0.38–0.50). The DOR (Fig. 3E) was 4.62 (95%CI 3.76–5.68). Subsequently, we plotted the SROC curve (Fig. 3G), and the AUC was 0.72 (95%CI 0.68–0.75). These results suggest that tsRNAs have good efficacy in diagnosis of BC and have potential to be used as diagnostic markers. Moreover, bivariable box diagram results (Fig. 4A) showed that most studies were distributed within the 95%CI. To analyze sources of heterogeneity among the studies, we evaluated threshold effects. The results showed that the SROC curve graph (Fig. 3F) did not show a typical "shoulder-arm" distribution, considering that there was no threshold effect. Covariables country, expression, case, and type were used for subgroup analysis (Table 3) and meta-regression analysis (Table 4) to evaluate the nonthreshold effects. The *I*^2^ of the DOR for country (China vs. Japan) was 53.5% vs. 0.0%, and the *I*^2^ of the DOR for expression (Down vs. Up vs. NA) was 62.9% vs. 40.7% vs. 0.0%, partially reducing heterogeneity. However, in univariate multiple regression analysis, the covariables were all *P* > 0.05, no significant correlation was found with DOR, and heterogeneity due to experimental design was not considered.

**Table 2:** Characteristics of all diagnostic studies included in the meta-analysis

No	Study	Year	Country	Expre ssion	Sample size		Variables										Cancer type	Ref^k^
					Case	Control	AUC^a^	Sen^b^	Spe^c^	TP^d^	FP^e^	TN^f^	FN^g^	PLR^h^	NLR^i^	DOR^j^		
1	Mo, 5′-tiRNA-Val	2019	China	Down	60	20	0.756	0.900	0.627	54	7	13	6	2.413	0.159	15.129	BC^l^	31078732
2	Huang, tDR‐7816	2019	China	Down	45	22	0.859	0.832	0.709	37	6	16	8	2.855	0.237	12.046	NTNBC^m^	31535382
3	Huang, tDR − 5236	2019	China	NA	45	22	0.588	0.729	0.539	33	10	12	12	1.579	0.504	3.131	NTNBC	31535382
4	Huang, tDR − 5334	2019	China	Down	45	22	0.661	0.730	0.611	33	9	13	12	1.873	0.443	4.229	NTNBC	31535382
5	Huang, tDR − 4733	2019	China	Down	45	22	0.621	0.627	0.609	28	9	13	17	1.602	0.613	2.613	NTNBC	31535382
6	Huang, tDR − 6954	2019	China	NA	45	22	0.567	0.615	0.533	28	10	12	17	1.317	0.722	1.823	NTNBC	31535382
7	Koi, tRF-Lys (TTT)	2019	Japan	Up	39	36	0.773	0.744	0.806	29	7	29	10	3.835	0.318	12.074	BC	32215990
8	Koi, tRF-Lys (TTT)	2019	Japan	Up	39	36	0.720	0.718	0.722	28	10	26	11	2.583	0.391	6.613	BC	32215990
9	Wang, tRF-Glu-CTC-003	2020	China	Down	144	112	0.684	0.659	0.617	95	43	69	49	1.721	0.552	3.115	EBCn	32814252
10	Wang, tRF-Gly-CCC-007	2020	China	Down	144	112	0.758	0.653	0.739	94	29	83	50	2.504	0.469	5.340	EBC	32814252
11	Wang, tRF-Gly-CCC-008	2020	China	Down	144	112	0.630	0.596	0.533	86	52	60	58	1.275	0.759	1.680	EBC	32814252
12	Wang, tRF-Leu-CAA-003	2020	China	Down	144	112	0.772	0.723	0.705	104	33	79	40	2.453	0.392	6.250	EBC	32814252
13	Wang, tRF-Ser-TGA-001	2020	China	Down	144	112	0.740	0.752	0.613	108	43	69	36	1.942	0.405	4.794	EBC	32814252
14	Wang, tRF-Ser-TGA-002	2020	China	Down	144	112	0.739	0.749	0.651	108	39	73	36	2.148	0.386	5.571	EBC	32814252
15	Wang, tRF-Arg-CCT-017	2021	China	Up	120	112	0.683	0.685	0.617	82	43	69	38	1.786	0.511	3.496	BC	33402674
16	Wang, tRF-Gly-CCC-001	2021	China	Up	120	112	0.656	0.622	0.657	75	38	74	45	1.813	0.575	3.153	BC	33402674
17	Wang, tiRNA-Phe-GAA-003	2021	China	Up	120	112	0.666	0.552	0.749	66	28	84	54	2.198	0.598	3.674	BC	33402674
18	Mo, tRF-17-79MP9PP	2021	China	Down	76	27	0.750	0.704	0.684	54	9	18	22	2.228	0.433	5.148	BC	33912465
19	Zhang, tRF-Gly-CCC-046	2021	China	Up	214	113	0.722	0.804	0.558	172	50	63	42	1.819	0.351	5.179	BC	34254739
20	Zhang, tRF-Tyr-GTA-010	2021	China	Up	214	113	0.781	0.836	0.619	179	43	70	35	2.194	0.265	8.282	BC	34254739
21	Zhang, tRF-Pro-TGG-001	2021	China	Up	214	113	0.709	0.822	0.531	176	53	60	38	1.753	0.335	5.229	BC	34254739

a AUC: the area under the receiver operating characteristic curve; b Sen: sensitivity; c Spe: specificity; d TP: true positive; e FP: false positive; f TN: true negative; g FN: false negative; h PLR: positive likelihood ratio; i NLR: negative likelihood ratio; j DOR: diagnostic odds ratio; k Ref: reference; l BC: breast cancer; m NTNBC: non-triple negative breast cancer; n EBC: early-stage breast cancer

**Table 3:** Assessment of diagnostic accuracy and heterogeneity in subgroup analysis

Category	Number of studies	Sensitivity	Specificity	Positive Likelihood Ratio	Negative Likelihood Ratio	Diagnostic Odds Ratio	*I*^2^ (%) of DOR
Total	21	0.72 (0.71–0.74)	0.64 (0.61–0.66)	1.94 (1.76–2.12)	0.44 (0.38–0.51)	4.55 (3.67- 5.65)	52.9
Country							
China	19	0.72 (0.71–0.74)	0.63 (0.61–0.66)	1.90 (1.74–2.07)	0.45 (0.39–0.52)	4.37 (3.51–5.44)	53.3
Japan	2	0.73 (0.62–0.83)	0.76 (0.65–0.86)	3.02 (1.95–4.67)	0.35 (0.24–0.52)	8.70 (4.15–18.28)	0.0
Expression							
Down	11	0.71 (0.68–0.73)	0.65 (0.61–0.68)	1.97 (1.68–2.31)	0.45 (0.37–0.54)	4.55 (3.22–6.43)	62.9
Up	8	0.75 (0.72–0.77)	0.64 (0.60–0.67)	1.95 (1.75–2.18)	0.41 (0.33–0.52)	4.97 (3.77–6.56)	40.7
NA	2	0.68 (0.57–0.77)	0.55 (0.39–0.70)	1.49 (1.05–2.12)	0.60 (0.40–0.89)	2.53 (1.21–5.32)	0.0
Case							
> 100	12	0.72 (0.70–0.74)	0.63 (0.61–0.66)	1.91 (1.72–2.12)	0.45 (0.39–0.54)	4.33 (3.40–5.50)	59.7
< 100	9	0.74 (0.69–0.78)	0.66 (0.60–0.73)	2.05 (1.65–2.54)	0.41 (0.31–0.54)	5.31 (3.29–8.57)	44.0
Type							
BC^a^	10	0.75 (0.73–0.78)	0.64 (0.60–0.67)	1.96 (1.78–2.16)	0.39 (0.32–0.49)	5.23 (3.98–6.89)	41.8
EBC^b^	6	0.69 (0.66–0.72)	0.64 (0.61–0.68)	1.94 (1.57–2.39)	0.48 (0.39–0.60)	4.08 (2.71–6.12)	71.7
NTNBC^**c**^	5	0.71 (0.64–0.77)	0.60 (0.50–0.69)	1.70 (1.33–2.16)	0.49 (0.35–0.69)	3.59 (1.99–6.48)	32.5

a BC: breast cancer; b EBC: early-stage breast cancer; c NTNBC: non-triple negative breast cancer

**Table 4:** Results of univariate meta-regression analysis of diagnostic odds ratio

Covariables	*P*-value	RDOR^a^	95%CI
Country (China/Japan)	0.118	2.43	(0.78–7.60)
Expression (Down/Up/NA)	0.123	0.68	(0.41–1.12)
Case (> 100/ < 100)	0.870	1.05	(0.58–1.88)
Type (BC/EBC/NTNBC^b^)	0.189	0.80	(0.57–1.13)

a RDOR: relative DOR; b BC/EBC/NTNBC: breast cancer/early-stage breast cancer/non-triple negative breast cancer

To further evaluate the ability of tsRNAs as diagnostic markers in patients with BC, Fagan's nomogram (Fig. 4C) and Scatter plots of PLR and NLR (Fig. 4D) were developed. When the prior probability is 20%, the probability of BC in positive tsRNAs test increases to 33%, and the probability of BC in negative tsRNAs test decreases to 10%. All the above results indicate that tsRNAs have high diagnostic capability of BC and are a good diagnostic test.

### Clinicopathological roles of tsRNAs in BC

**Fig. 5:**
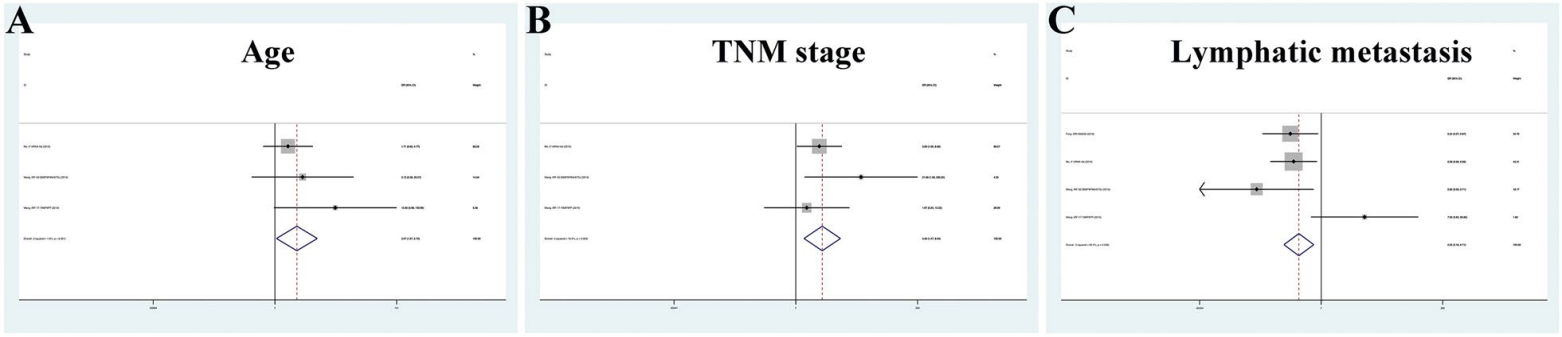
Forest plots of age (**A**), TNM stage (**B**) and lymphatic metastasis (**C**) of tsRNAs in breast cancer

A total of 3 studies examining 5 types of tsRNAs included clinicopathological data (Table 5) [13, 25, 28]. Considering the heterogeneity among different studies, we only summarized the studies with at least 3 relevant indicators. The results showed that tDR-000620, 5'-tiRNA-Val, tRF-32-Q99P9P9NH57SJ, tRF-17-79MP9PP were all expressed at low levels in BC patients and correlated with patient age (OR = 2.470, 95%CI 1.067–5.718), TNM stage (OR = 3.435, 95%CI 1.468–8.039), lymphatic metastasis (OR = 0.354, 95%CI 0.177–0.709) (Fig. 5). Regarding the correlation between other indicators and tsRNAs, more studies need to be evaluated for confirmation.

**Table 5:** Meta-analyses of correlation between downregulated tsRNAs and clinicopathological features of breast cancer

Downregulated tsRNAs	No. of studies	No. of patients	Odds ratio (95%CI)	*p*-value	Heterogeneity	*I*^2^ (%)
Age (≤ 50/ > 50)	3	92	2.470 (1.067–5.718)	0.361	2.04	1.8
TNM stage (I-II/III-IV)	3	92	3.435 (1.468–8.039)	0.304	2.38	16.0
Lymphatic metastasis ( ±)	4	136	0.354 (0.177–0.709)	0.038	8.42	64.4

### Sensitivity analysis and publication bias

To assess the impact of each study on the results of the meta-analysis, sensitivity analysis was performed on the included literature. The results showed no significant change in the combined total HRs or ORs after the removal of each study, suggesting little difference among studies and relatively stable results (Additional file 4: Figure S4 and Additional file 5: Figure S5).

Begg’s funnel plots and the Egger test were used to assess the existence of publication bias. The funnel plots of prognostic correlation studies were roughly symmetric (Additional file 1: Figure S4). However, one of the lymphatic metastasis indexes in the clinicopathological feature correlation analysis fell outside the funnel plot, suggesting publication bias in the included studies, which may be related to the small amount of data (Additional file 5: Figure S5). Finally, Deeks’ funnel plots were applied to analyze publication bias in diagnostic correlation studies. The results showed a basically symmetrical funnel plot (*P* = 0.59), suggesting no significant publication bias (Fig. 4B).

## Discussion

BC is the malignant tumor with the highest incidence in the female population at present [35, 36]. Its causes are still unclear, and some types lack effective intervention targets, resulting in a high fatality rate. Traditional BC detection methods, such as CEA, CA153, B-ultrasound, and molybdenum targeted therapy, among others, are relatively less traumatic than other methods but have low sensitivity and specificity for early diagnosis, and pathology, the gold standard of diagnosis, is difficult to popularize in the population without disease [37–40]. Therefore, exploration of more effective markers for early diagnosis and precise treatment is crucial for improving the poor prognosis of BC patients.

As emerging ncRNAs, abnormal expression of tsRNAs has been confirmed in a variety of diseases, including cancers, and plays an important biological role and function [41, 42]. As tRNA fragments, tsRNAs are characterized by low molecular weight and stable expression. In addition, tsRNAs are widely expressed and specific in human tissues and body fluids. These characteristics all render tsRNAs important measures in cancer diagnosis and prognosis prediction [43, 44]. Since Hani et al. discovered that tRFs derived from tRNA-Glu, tRNA-Asp, tRNA-Gly, and tRNA-Tyr competitively bind to the endogenous oncogene YBX1 in BC cells, inhibiting cell growth by interfering with oncogene transcription stability, the role and mechanism of tsRNAs in BC occurrence and development have been extensively explored [15]. Wang et al. conducted tsRNA expression sequencing on plasma samples from 8 BC patients and 4 healthy women, and found that the expression level of tRF-Glu-CTC-003 in BC patients' plasma was lower compared to that in healthy women, and the expression level of tRF-Glu-CTC-003 in TNBC patients' plasma was lower than in other subtypes [31]. These differential results suggest that tsRNA may serve as a potential biomarker for BC. Detecting tsRNA levels in plasma can aid in early BC diagnosis and patient prognosis evaluation. Mechanistically, tsRNAs primarily regulate protein expression at different stages by binding to other molecules, thereby affecting protein biosynthesis through transcription or post-transcriptional processes, and playing a regulatory role in BC. Maurizio et al. found significantly lower expression levels of tRF3E derived from mature tRNA-Glu in BC tissues compared to normal tissues, which could inhibit BC progression by binding to the RNA-binding protein NCL [45]. Zhu et al. identified high expression of tRF-Lys-CTT-010 in TNBC, demonstrating its ability to promote cell proliferation and migration, participate in metabolic pathways, and regulate cell survival and proliferation by manipulating lactic acid production and glycogen consumption [46]. Mo et al. discovered the inhibitory effects of tRF-17-79MP9PP on BC cell invasion and metastasis via the THBS1/TGF-β1/Smad3 axis [30]. These findings collectively indicate that tsRNA plays a crucial role in BC occurrence and development, offering potential avenues for precise BC treatment.

To our knowledge, this is the first meta-analysis exploring the value of tsRNAs in BC, with a view to providing evidence-based medical evidence for future clinical applications of tsRNAs. According to our inclusion and exclusion criteria, 13 studies on the correlation between tsRNAs and BC were identified as eligible for meta-analysis, and no studies that did not meet the criteria were found after quality assessment. In the study on the correlation between tsRNAs and BC patient prognosis, we included 7 studies involving a total of 5257 patients. The results showed that tsRNA expression correlated positively with prognostic indicators (PFS, RFS, DFS, OS), which was consistent with the study on the mechanism of differentially expressed tsRNAs playing a role in promoting or suppressing BC. It should be noted that the cut-off method for classifying tsRNA expression was not unified among the included studies, and there is still a lack of quantitative analysis standards for tsRNAs, posing challenges for practical clinical application of tsRNAs in the future. In addition, the vast majority of studies focused on Asia, and more solid research is needed to confirm whether our results are biased by race and/or region. Wang et al. found that the AUC of the combination of six tsRNAs in the plasma of early-stage BC patients was 0.844, superior to any single identified tsRNA [32]. In addition, Zhang et al. reported that when three tsRNAs were combined with the traditional tumor markers CEA, CA125 and CA153, the AUC increased to 0.801 [33]. These results suggest that tsRNAs have reliable potential for BC diagnosis. Therefore, we systematically reviewed studies on the diagnostic value of tsRNAs in BC, aiming to identify biomarkers that can be used for diagnosis. Eventually, 7 studies involving 21 different tsRNAs were included in our analysis. After meta-analysis of all tsRNAs, the combined SEN was 72% (95%CI 68–76%), the combined SEP was 64% (95%CI 61–67%), and the AUC of the SROC curve was 0.72 (95%CI 0.68–0.75), indicating that tsRNAs have good diagnostic efficacy. As an independent prognostic indicator, the DOR value can indicate the degree of association between diagnosis results and diseases, whereby a higher value indicates a more reliable diagnosis. In diagnostic assessment of tsRNAs, the combined DOR reached 4.62 (95 CI 3.76–5.68), further indicating reliable accuracy of tsRNA diagnosis in BC. Finally, Fagan's nomogram was used to analyze the clinical value of tsRNAs. The results showed that when the pretest probability was set to 20%, the probability of BC in positive tsRNA test results increased to 30% and that in negative tsRNA test results decreased to 10%. Regarding clinicopathological studies, 3 studies were included in total. The results showed that low expression of tsRNAs correlated significantly with age, TNM stage and lymphatic metastasis, suggesting a role in inhibiting BC progression. In addition, we found a certain publication bias in the included studies, which affected the accuracy of the results obtained; the reason may be that there were few studies and that some of them had small sample sizes. For example, in the study of Wang et al. ts-32-Q99P9P9NH57SJ and ts-17-79MP9PP showed low expression in BC, though the trend in lymphatic metastasis was the opposite, which may also be related to the small number of samples (*n* = 16) [28]. In conclusion, we believe that tsRNAs may become a new target in BC treatment and provide new ideas for BC treatment. However, more literature needs to be examined to obtain reliable data support.

At present, research on tsRNAs is still in the initial stage, and the specific molecular mechanism of the role of a large number of differentially expressed tsRNAs in tumors, especially BC, has not been clarified. Nevertheless, we hope that our meta-analysis will be helpful for future clinical applications of tsRNAs. Of course, there are several limitations of this study that should not be ignored. First, as the patients included were mostly from China, the conclusion may not be applicable to different regions or populations. Second, some data and their 95%CIs were obtained indirectly through software, which may lead to deviation from the real data, resulting in certain publication bias. In addition, there are few related studies on different subtypes, especially triple-negative breast cancer; thus, the positive role of tsRNAs in the early diagnosis and prognosis of these patients should be explored. Finally, comprehensive treatment tolerance is one of the main factors affecting the survival of BC patients at present, but there are few studies focusing on such factors. Future studies with more and larger samples should be designed and performed to guide individualized clinical therapy (Additional file 6: Figure S6).

## Conclusion

In summary, our study found that tsRNAs have important value in diagnosis and prognosis assessment of BC and correlate significantly with some clinicopathological features, suggesting that tsRNAs can be used as an effective diagnostic and treatment marker for BC patients. However, due to the limited sample size, the results may be unstable. Therefore, more high-quality research data are needed to support or update our conclusions in the future.

### Supplementary Information

**Additional file 1 Figure S1.** NOS risk of bias assessment.

**Additional file 2 Figure S2.** QUADAS 2 risk of bias assessment.

**Additional file 3 Figure S3.** Subgroup analyses for upregulated tsRNAs in breast cancer, including country (**A**), cut-off (**B**), sample size (**C**), data source (**D**), detected sample (**E**), follow-up time (**F**), survival outcome (**G**), survival analysis (**H**) and HR obtained (**I**)

**Additional file 4 Figure S4.** Subgroup analyses for downregulated tsRNAs in breast cancer, including cut-off (**A**), sample size (**B**), data source (**C**), detected sample (**D**), follow-up time (**E**), survival outcome (**F**), survival analysis (**G**) and HR obtained (**H**).

**Additional file 5 Figure S5.** Sensitivity analysis (**A** and **D**), publication bias judged by Begg’s (**B** and **E**) and Egger’s (**C** and **F**) funnel plots of tsRNAs for survival outcome of breast cancer.

**Additional file 6 Figure S6.** Sensitivity analysis (**A**, **D** and **G**), publication bias judged by Begg’s (**B**, **E** and **H**) and Egger’s (**C**, **F** and **I**) funnel plots of tsRNAs for age, TNM stage and lymphatic metastasis of breast cancer.

## Data Availability

All data generated or analyzed during this study are included in this published article.

## Abbreviations

BC: Breast cancer
ncRNA: Non-coding RNA
miRNA: Micro RNA
lncRNA: Long non-coding RNA
circRNA: Circular RNA
tsRNA: TRNA-derived small RNA
tRF: TRNA-related fragment
tiRNA: TRNA halve
PFS: Progression-free survival
RFS: Recurrence-free survival
DFS: Disease-free survival
OS: Overall survival
HR: Hazard ratio
95%CI: 95% Confidence interval
AUC: Area under the curve
SEN: Sensitivity
SPE: Specificity
TP: True positive
FP: False positive
TN: True negative
FN: False negative
PLR: Positive likelihood ratio
NLR: Negative likelihood ratio
DOR: Diagnostic odds ratio
NOS: Newcastle–ottawa score
QUADAS 2: Quality assessment for studies of diagnostic accuracy 2
OR: Odds ratio
SROC: Summary receiver operator characteristic
