# Supplementary figures and images for: Prognostic, diagnostic and clinicopathological roles of tsRNAs: a meta-analysis in breast cancer
Source: Eur J Med Res. 2024 Jan 8;29:35. doi: 10.1186/s40001-023-01617-2 (PMC10773143; doi:10.1186/s40001-023-01617-2)

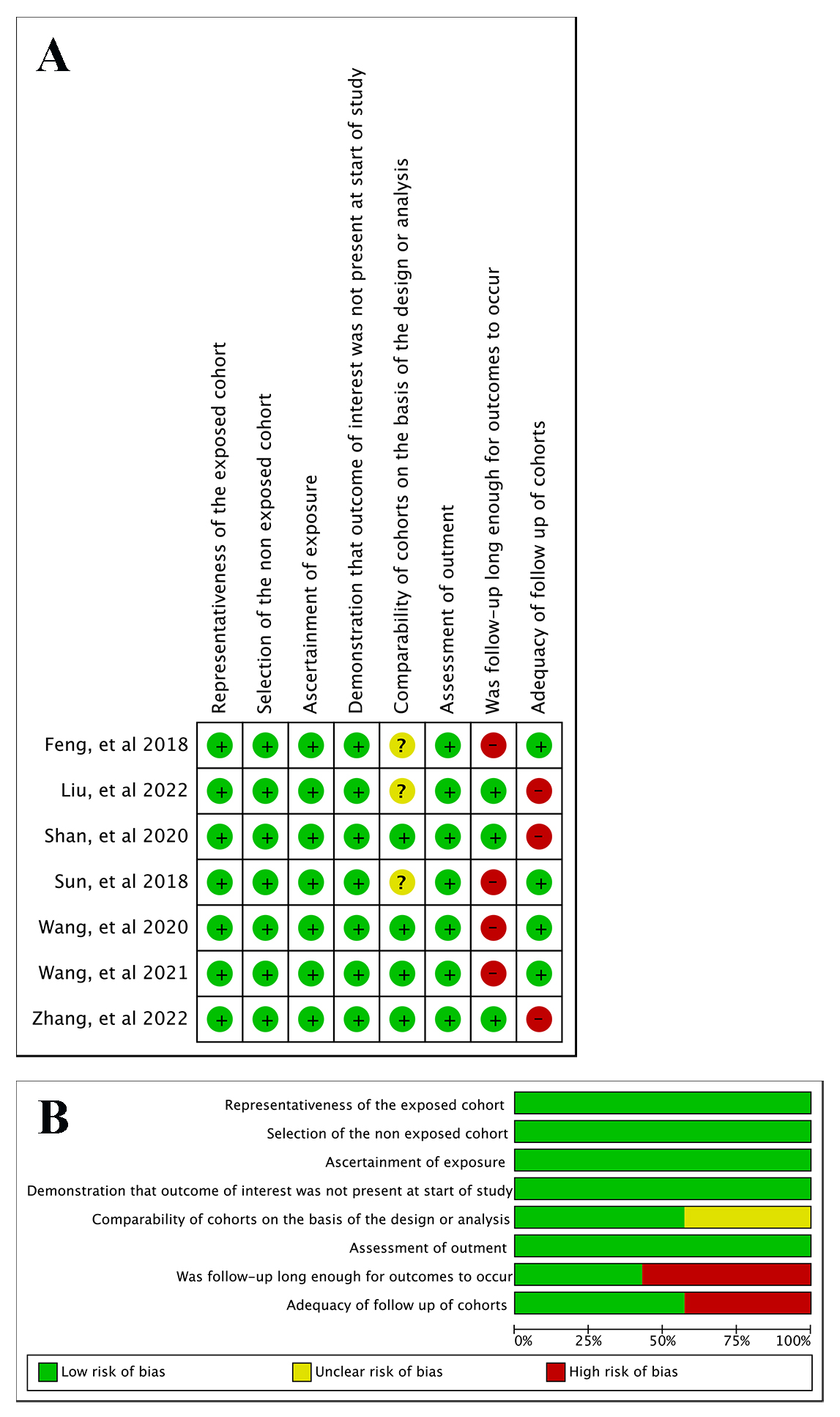

Supplement: Supplementary file 1 — Additional file 1 Figure S1. NOS risk of bias assessment. [file 40001_2023_1617_MOESM1_ESM.jpg]

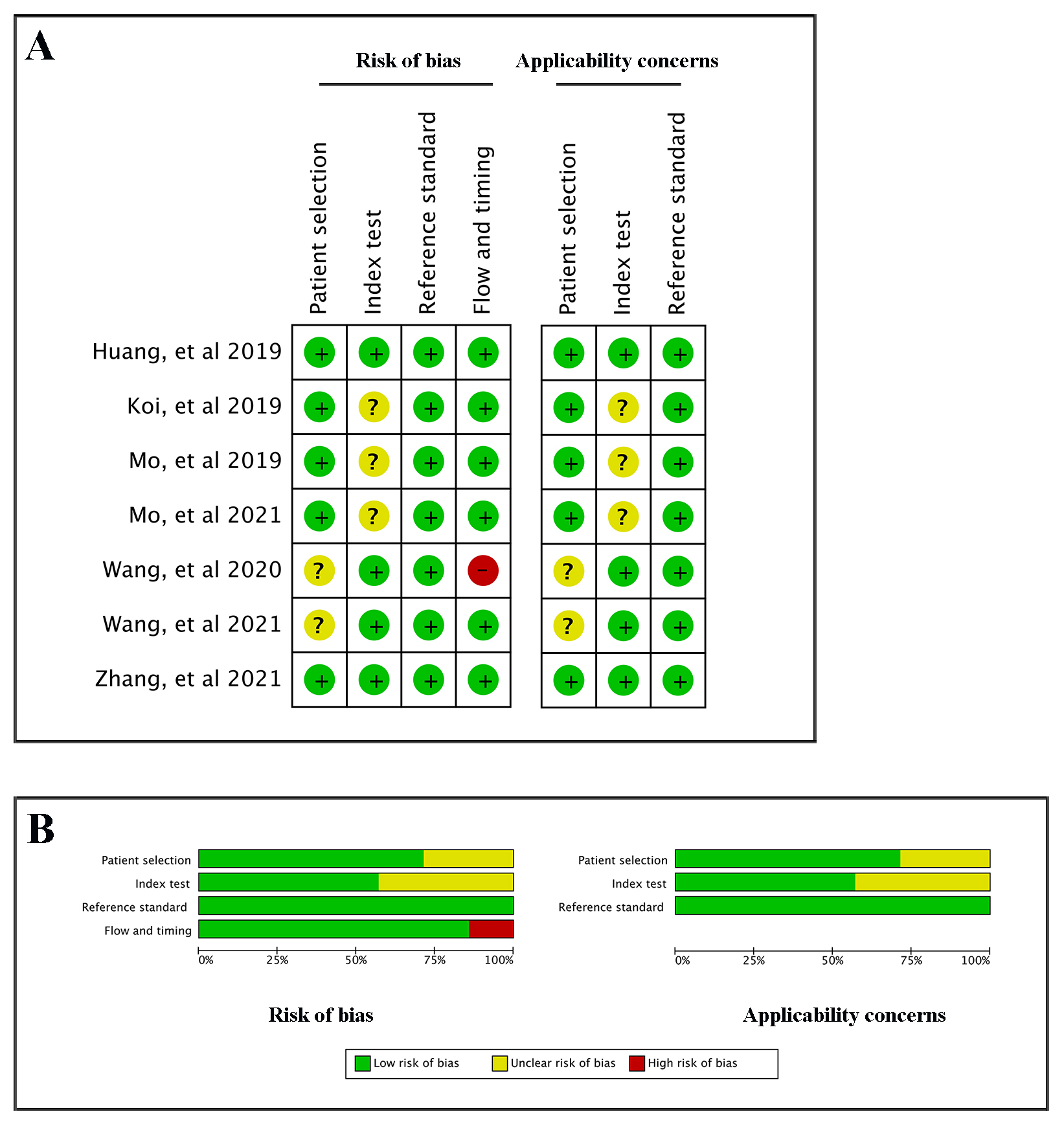

Supplement: Supplementary file 2 — Additional file 2 Figure S2. QUADAS 2 risk of bias assessment. [file 40001_2023_1617_MOESM2_ESM.jpg]

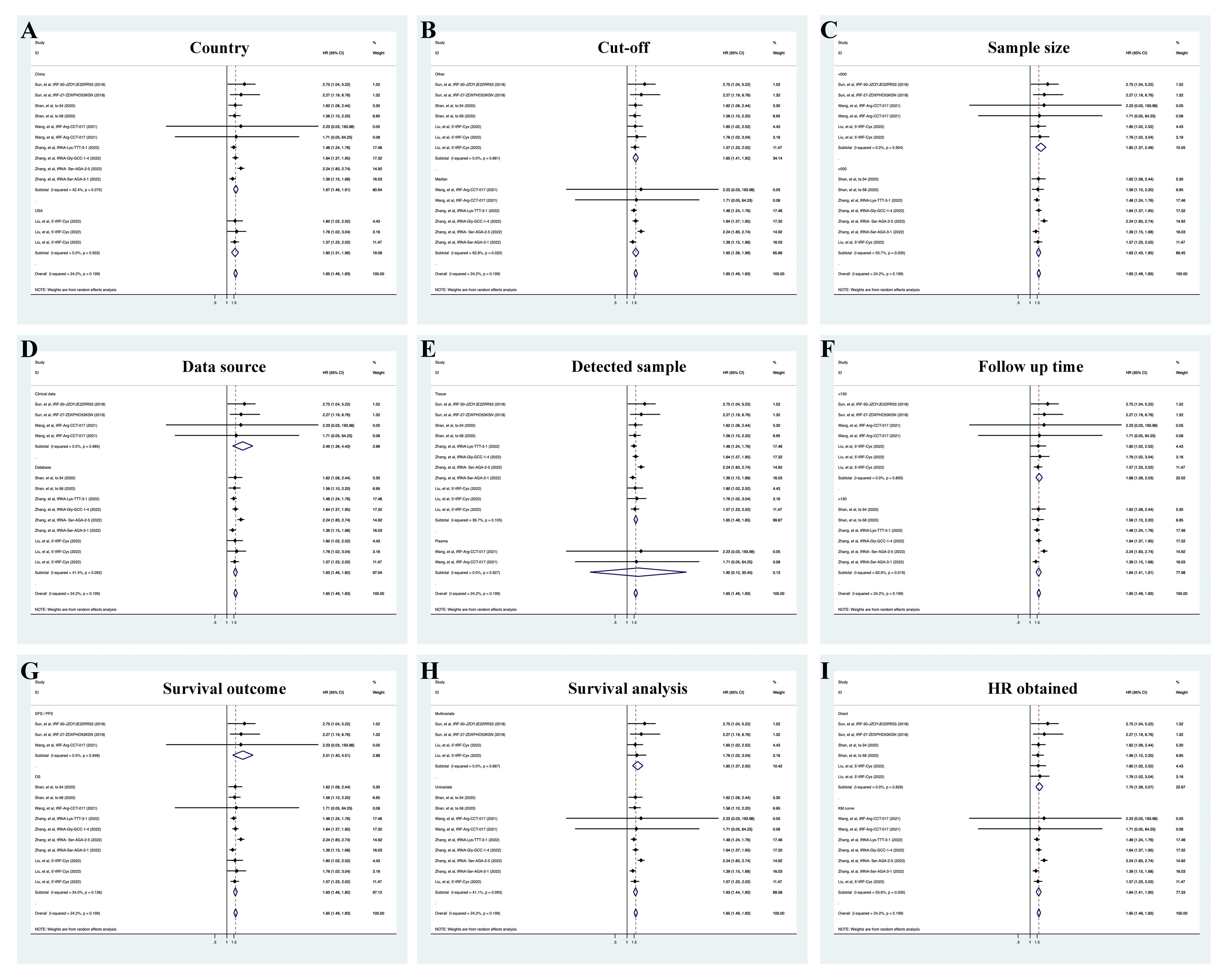

Supplement: Supplementary file 3 — Additional file 3 Figure S3. Subgroup analyses for upregulated tsRNAs in breast cancer, including country (A), cut-off (B), sample size (C), data source (D), detected sample (E), follow-up time (F), survival outcome (G), survival analysis (H) and HR obtained (I) [file 40001_2023_1617_MOESM3_ESM.jpg]

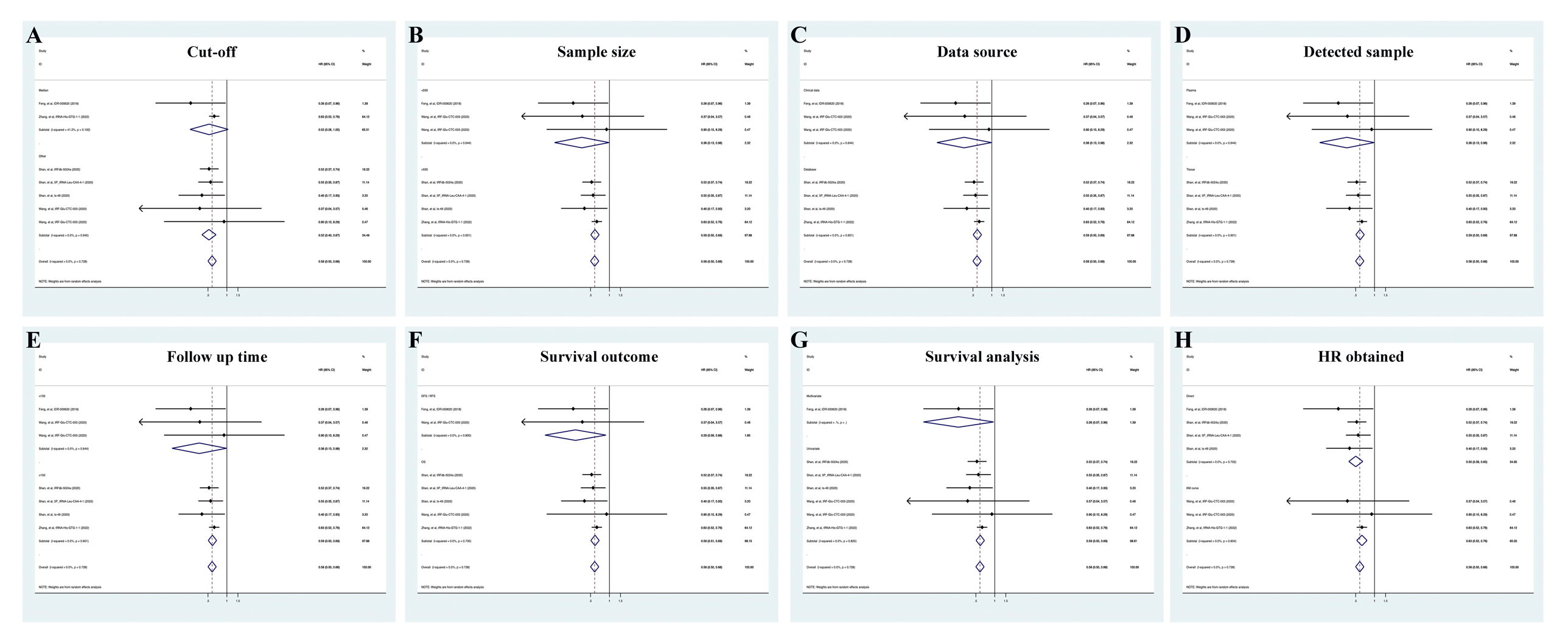

Supplement: Supplementary file 4 — Additional file 4 Figure S4. Subgroup analyses for downregulated tsRNAs in breast cancer, including cut-off (A), sample size (B), data source (C), detected sample (D), follow-up time (E), survival outcome (F), survival analysis (G) and HR obtained (H). [file 40001_2023_1617_MOESM4_ESM.jpg]

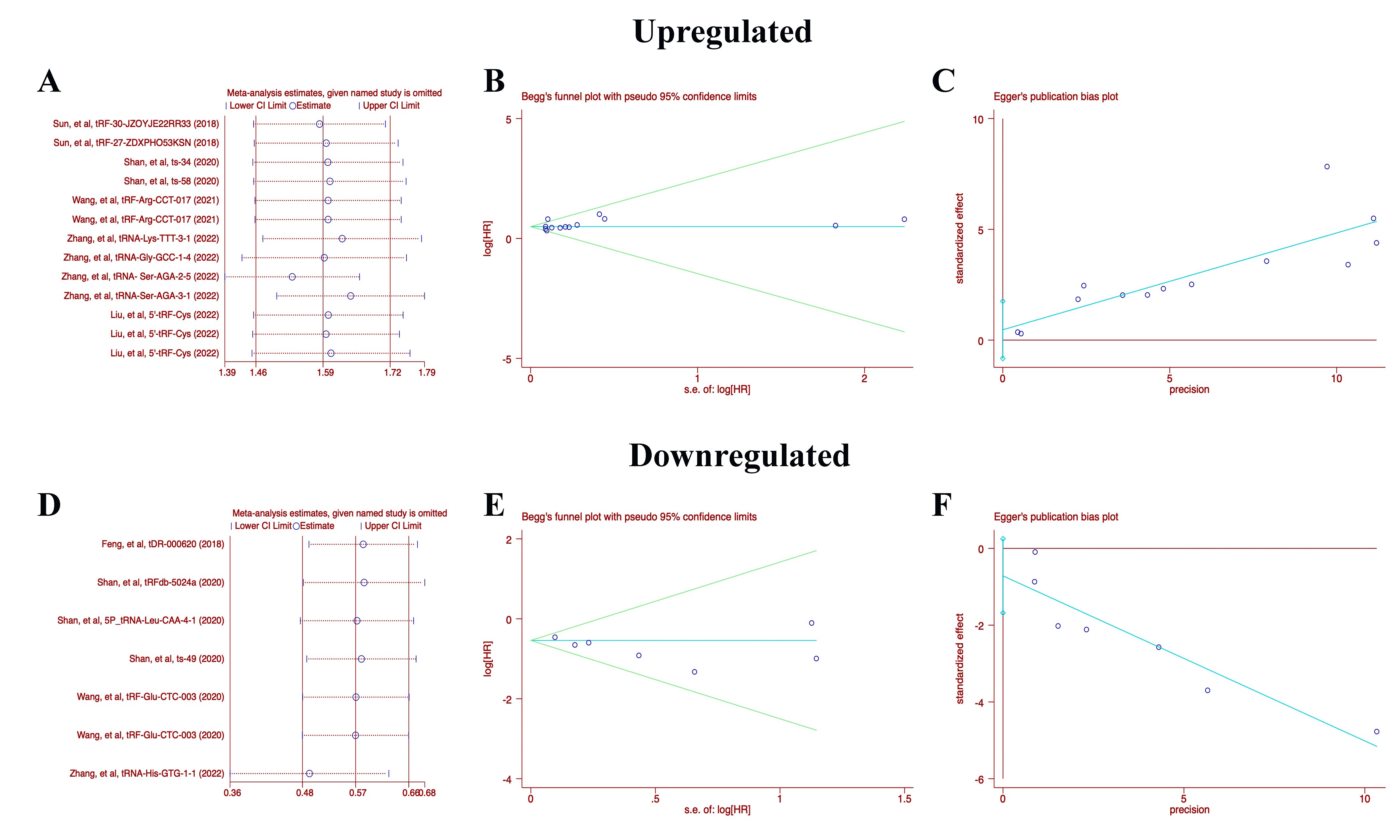

Supplement: Supplementary file 5 — Additional file 5 Figure S5. Sensitivity analysis (A and D), publication bias judged by Begg’s (B and E) and Egger’s (C and F) funnel plots of tsRNAs for survival outcome of breast cancer. [file 40001_2023_1617_MOESM5_ESM.jpg]

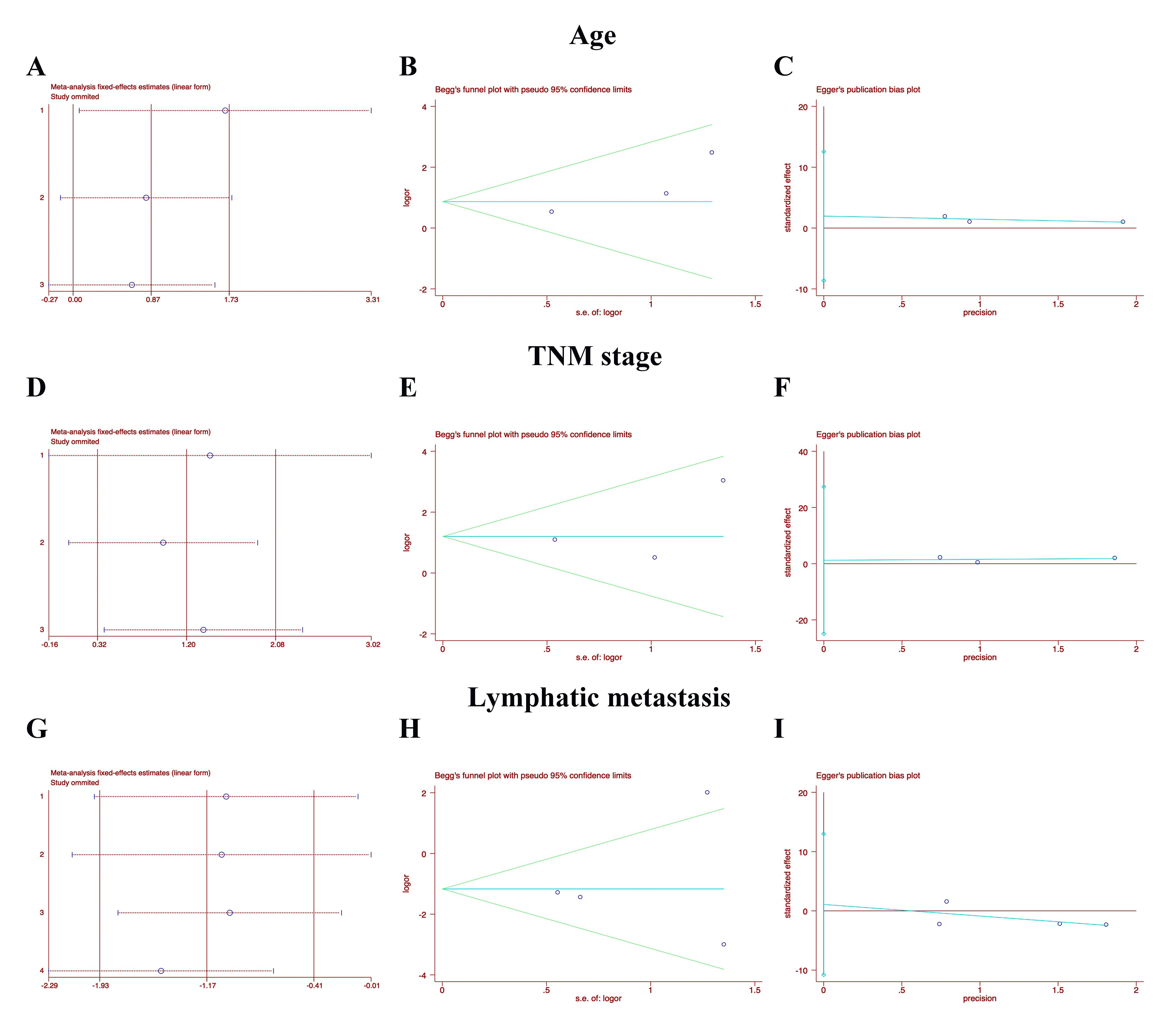

Supplement: Supplementary file 6 — Additional file 6 Figure S6. Sensitivity analysis (A, D and G), publication bias judged by Begg’s (B, E and H) and Egger’s (C, F and I) funnel plots of tsRNAs for age, TNM stage and lymphatic metastasis of breast cancer. [file 40001_2023_1617_MOESM6_ESM.jpg]
